# Fast *k*-NNG Construction with GPU-Based Quick Multi-Select

**DOI:** 10.1371/journal.pone.0092409

**Published:** 2014-05-08

**Authors:** Ivan Komarov, Ali Dashti, Roshan M. D'Souza

**Affiliations:** Department of Mechanical Engineering, Complex Systems Simulation Lab, University of Wisconsin-Milwaukee, Milwaukee, Wisconsin, United States of America; University of Vigo, Spain

## Abstract

In this paper, we describe a new brute force algorithm for building the 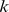-Nearest Neighbor Graph (*k*-NNG). The *k*-NNG algorithm has many applications in areas such as machine learning, bio-informatics, and clustering analysis. While there are very efficient algorithms for data of low dimensions, for high dimensional data the brute force search is the best algorithm. There are two main parts to the algorithm: the first part is finding the distances between the input vectors, which may be formulated as a matrix multiplication problem; the second is the selection of the *k*-NNs for each of the query vectors. For the second part, we describe a novel graphics processing unit (GPU)-based multi-select algorithm based on quick sort. Our optimization makes clever use of warp voting functions available on the latest GPUs along with user-controlled cache. Benchmarks show significant improvement over state-of-the-art implementations of the *k*-NN search on GPUs.

## Introduction

**Figure 1:**
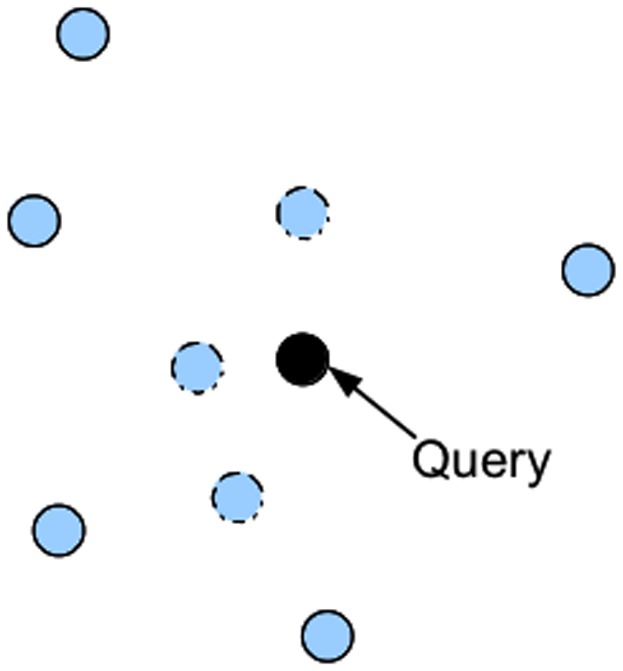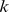-NN search. The blue dots are corpus data vectors. The blue dots with dashed lines are the 3 nearest neighbors to the query.

The *k*-nearest neighbor (*k*-NN) (illustrated in Figure 1) and the related *k*-nearest neighbor graph (*k*-NNG) are important algorithms for data classification with a wide variety of applications in areas such as bioinformatics [1], [2], data mining [3], machine learning [4]–[6], cluster analysis [7], and pattern recognition [8]. Given an initial set of data points called a corpus/training set and a set of query points, *k*-NN finds the nearest 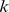 neighbors in the corpus set for each member of the query set. For low dimensional data-sets, there are a variety of indexing data structures such as kd-trees [9], BBD-trees [10], random-projection trees (rp-trees) [11], and hashing based on locally sensitive hash [12] that can be built from the set of corpus/training data points. Next, the *k*-NN search can be accomplished very efficiently for each data point in the query set using these data structures. In the case of a 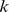-NNG, every data point in the corpus is also a member of the query set.

Direct construction of an exact *k*-NNG has also been investigated by many researchers [13]–[18]. All of these methods have time complexity that increases exponentially with the data dimension. Approximate methods that can handle high-dimensional data with a trade-off between speed and accuracy have also been investigated. These techniques are based on a hybrid of spatial subdivision and small scale brute force refinement [19]–[21]. The spatial subdivision becomes grossly inaccurate for data sets that have dimensions exceeding 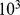.

For high dimensional data, the most efficient method for finding the *k*-NNG is in fact the brute force method [22]. The brute force algorithm consists of two fundamental steps. The first step finds the distances between the query point based on an user-defined metric. This process result in a distance matrix of size 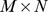 where 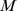 is the number of query points and 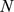 is the number of corpus data points. The 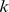-NNs in the corpus data points for each query points are then found by sorting each row of the matrix and finding the 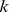 indices of the 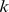 smallest distances. Recent efforts have accelerated the brute force 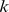-NN and 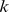-NNG methods by parallelizing on graphics processing units. These methods differ in the manner in which the 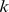 nearest indices are found. In [23], [24], each row of the distance matrix is processed by one thread. They use a modified insertion sort algorithm to select the 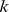 nearest neighbors. The data structure is stored in global memory, which in turn leads to un-coalesced memory access patterns. Moreover, each thread has divergent paths to find the 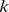 nearest neighbors, which leads to a loss in computational efficiency. In [25], every row in the distance matrix is processed by one thread block. A single heap in global memory maintains the current 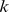 nearest elements. Each thread strides through the row of the matrix and inserts an element into a buffer if it is smaller that the largest element in the heap. When the buffer fills up, all threads synchronize and push their elements into the heap serially. In [26], a thread block is used to process a single row. Each thread in the thread block strides through the array storing the 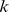 smallest elements in a local heap maintained in global memory. Next, all the thread heaps are merged into 32 heaps in shared memory threads in a single thread warp. Finally, a single thread merges the 32 heaps in shared memory to find the 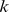 nearest neighbors. Both methods dramatically slow down if 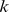 grows large because of branch divergence and un-coalesced global memory access. In [27], radix sort is used for finding 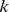 nearest neigbhors. Each row is processed in a separate kernel call and for the number 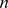 of data-corpus size that fits into GPU memory, this process underutilizes resources.

### Graphics Processing Units: Architecture and Execution Model

Graphics Processing Units (GPUs) were originally developed to handle computations related to computer graphics tasks such as transformation and shading. Computational scientists used this functionality to accelerate scientific computing by posing such computations in terms of shading operations [28]. Subsequently, GPU vendors developed specialized C language extensions to facilitate direct access to the computing hardware for scientific computing. Our implementation uses CUDA [29], a C language extension developed by NVIDIA.

Architecturally, an NVIDIA GPU consists of several multiprocessors (MPs). Each MP has several serial processors. In addition to registers, each MP has access to on-chip user-controlled cache called shared memory. Logically, threads are organized into thread blocks (TBs). All threads in a TB are executed on a single MP. Threads in a TB can be synchronized and can communicate through shared memory. Depending on the amount of shared memory and registers required, there could be several TBs assigned to be executed on a MP. All threads can communicate through global main memory. Registers have the highest access speed, followed by the shared memory, and then by global memory. Of course, data in the registers cannot be shared among threads. Shared memory locations are accessed through specific memory banks.

At the hardware level, threads in a TB are divided into groups of 32 threads called thread warps. Thread warps are analogous to the threads in a typical symmetric multiprocessor architecture. All threads in a thread warp execute in lockstep. Any branching between threads in a thread warp will cause serialization (different types of serialization depending on the complexity of the branching). Therefore it is advisable to avoid branching between threads of the same warp. During global memory access, if the threads in a warp access locations outside of contiguous 128 byte segments, the memory access will be serialized. Also, if two or more threads try to access shared memory affiliated to the same memory bank, this memory access will be serialized. An exception is when all threads in a warp access the same shared memory location. In such cases, a broadcast mechanism is automatically engaged. Therefore, implementations of algorithms for GPU acceleration require careful consideration of architectural limitations as well as advantages.

In our implementation we use an NVIDIA Tesla C2050 GPU. It has 14 multiprocessors, each with 32 cores, for a total of 448 cores. The single precision peak performance is 1.0 TFlops. Each MP has 64 KB of shared memory and a 128 KB register file. The main memory is 3 GB GDDR5.

## Methods and Materials

In this paper, we present a hybrid parallelization approach for brute force computation of multiple *k*-NN queries. The first part of the brute force *k*-NN, namely, finding the distance metric matrix, is formulated as a matrix multiplication problem. Therefore efficient implementations of dense matrix operations can be used. The second part is finding the *k*-NN for each query data point. For this part of brute force *k*-NN, we have developed a two-level, parallel implementation of the quick select algorithm. At the top or coarse level, multiple queries are processed independently and in parallel. At the fine level of parallelism, every query is parallelized over all threads in a thread block or thread warp.

### Related Work

Recently, there have been quite a few works focussed on parallel *k*-NN queries on GPUs. In [30], a truncated bitonic sort based selection algorithm has been described. The results show a dramatic drop off in performance with an increase in both the number of data points 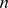 and number of nearest neighbors 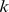. For example, the speedup over straight sort and selection using the CUDA thurst::sort for 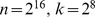, is barely 4X. In [31], a radix select implementation on the GPU is presented for selecting the 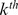 element. This implementation is very efficient for large values of 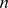 and runs up to 190X faster than a single core CPU implementation of the serial n-select algorithm. In [32], a randomized selection algorithm is presented. This method, however, shows no improvement over CUDA thrust::sort for data sizes of up to 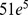. On the other hand, the implementation in [31] shows a 6.3X speedup. Both methods are however, only suited for single query operations on ultra-large data sets. A similar implementation has been presented by Alabi et al. [33]. The 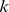-NN problem is slightly different from the method for selecting the 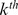 element because extra work is necessary to track all the 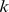 smallest elements.

In [34], a data-parallel quick-sort algorithm for GPUs has been presented. They describe a two stage process. In the first stage, all thread blocks cooperatively work on partitioning the original list to a point where the size of each partition is small enough to be processed by a single thread block. The first stage needs inter-thread block synchronization since the results of two or more thread blocks needs to be merged to build the partitions. This can be done through atomic operations or through default synchronization by ending kernel execution. The latter needs a new kernel launch for each new phase of partitioning. The second stage is very similar to the first, but each partition in this case is handled by a single thread block.

The partitioning process is a two step process and uses an auxiliary output array (i.e., it is not an in-place write operation). In the first step, a pivot element is randomly selected. Next, each thread strides through the input array counting the number of elements that are greater than, equal to, and less than the pivot. These two counts are stored in two arrays in shared memory. A pre-fix sum on these arrays in shared memory gives the location in global memory where each thread needs to write into the auxiliary the elements in the partitioning process. At the end of each partitioning task, the input and auxiliary arrays are swapped. When the partition size falls below a pre-defined threshold, a direct sort algorithm such as a bitonic sort is used.

### GPU-Based Quick Multi-Select

Our overall approach is illustrated in algorithm 1. Of the steps in the algorithm, distance computation and partitioning with pivot selection are done on the GPU by launching kernels. The decision process within the if-then-else statements are accomplished on the CPU. Each iteration of the while loop incurs a GPU kernel launch for partitioning with pivot selection. Our approach, while similar to the approach in [34], address some of the major drawbacks as related to the 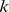-NN application: (1) the quick-sort algorithm in [34] is only suitable for sorting a single input array at a time; (2) inter thread block synchronization and synchronization between threads in the same thread block affects performance; (3) the pre-fix sum operation that is used during the partition process is quite an expensive operation; (4) the two step process of partitioning involves reading the same data from global memory twice (once for counting, once for writing); and (5) the write process into the auxiliary array is un-coalesced. Our approach, by contrast, operates on multiple arrays simultaneously. Each array is handled by a single thread warp. Threads in a warp are executed simultaneously on a single multi-processor are synchronized by default. Partitioning of an array is done incrementally in 32 element wide segments. We use shared memory to ensure coalesced memory writes of the results of partitioning into the auxiliary array in global memory. Our approach uses the warp voting function _ballot(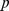) to partition the input without reading the input array twice and without executing the parallel-prefix sum. The ballot function _ballot(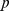) fills a 32 bit unsigned integer, one bit per thread in the warp, based on the evaluation of the predicate 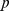 (see Algorithm 1 in Table 1).

**Table 1:** Algorithm 1: Overall data flow

**Data**: 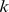 (number of nearest neighbors), 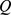 (set of query vectors), 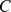 (set of data corpus vectors)
**Result:** Matrices of size 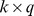 ( 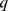 is the number of vectors in 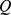 ) of the indices 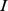 and values 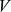 of 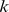 nearest vectors in 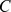 for each vector in 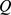
Compute the distance matrix 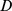 using matrix multiplication
Process for each row 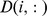 using either a warp or thread block
Set 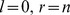
**While ** 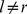 **do**
Select a pivot element 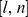
Partition 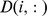 based on pivot
**if** 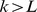 ( 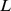 *is the length of the left side*) **then**
Set 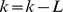
Set 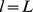 (keep the left side as is and partition the right side)
**else**
Set 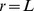 (discard the right side and partition the left side)
**end**
**end**

**Figure 2:**
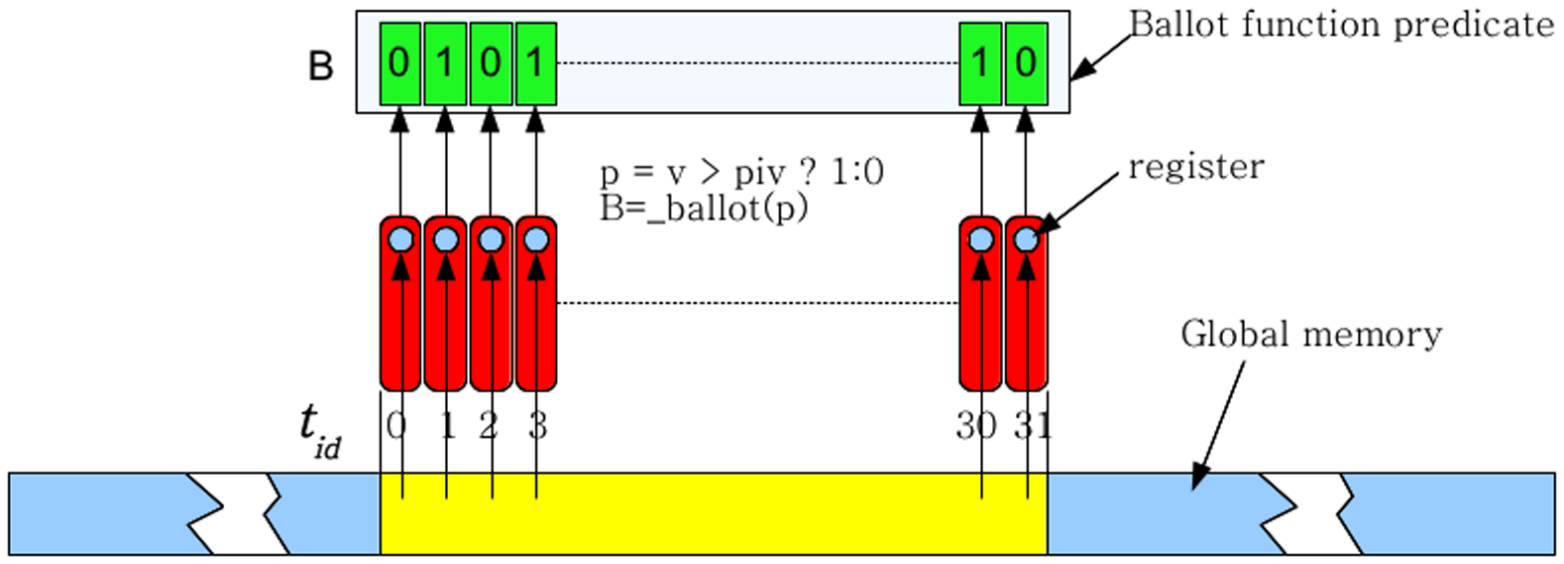
Read in process. Read in of the array is done incrementally in sets of 32 elements. As illustrated, the memory access is coalesced. The value is stored in a register. Simultaneously, the invocation of the warp voting function fills the bit array 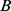 based on the evaluation of the predicate, which indicates if the value in the register is greater than or equal to the pivot or less than the pivot.

**Figure 3:**
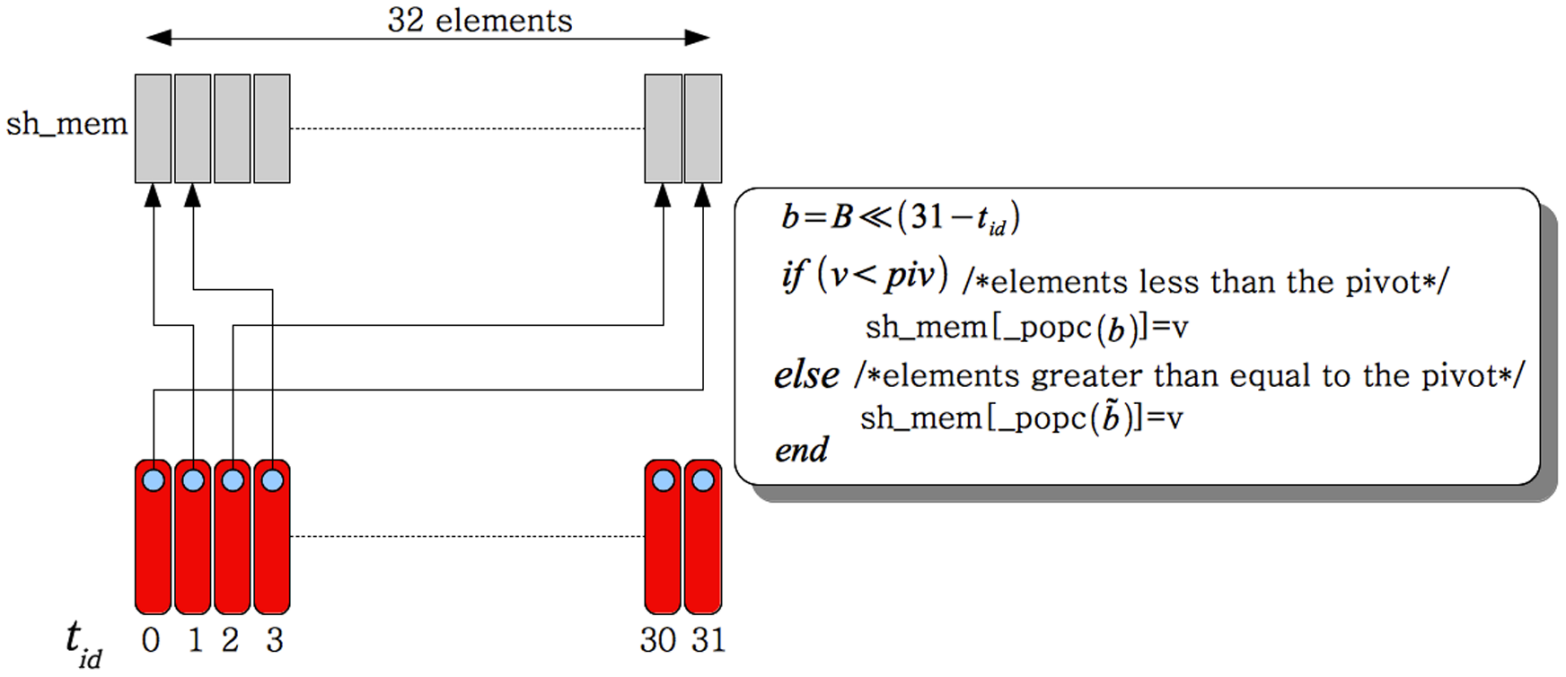
Pivot process. The pivot process is accomplished in shared memory. Each thread determines where in the shared memory the value has to be written. Values less than the pivot are accumulated on the left hand side and values greater than or equal to the pivot are accumulated on the right hand side. Since all threads write to different locations, there are no bank conflicts.

**Figure 4:**
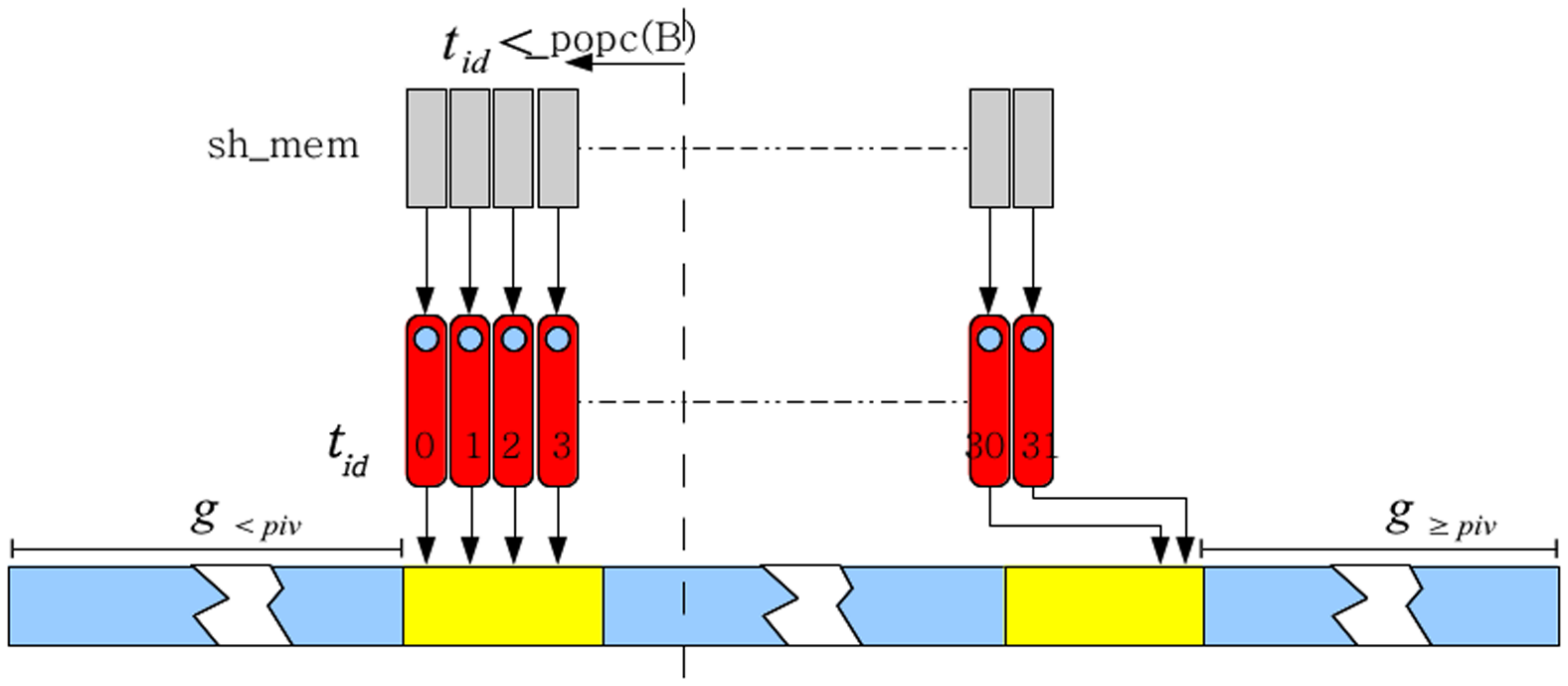
Write out process. The thread id indicates (based on the computation _popc(B)) whether a given thread is writing out an element less than the pivot or greater than or equal to the pivot. The values 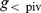 and 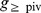, which are maintained in shared memory and updated incrementally, indicate the location in the global array of the location of the last element that is less than the pivot and greater than or equal to the pivot. This operation involves at most two coalesced memory writes.

Each thread in the warp first reads in an element into its register from global memory. Elements are then written to a 32 element wide shared memory array with elements greater than or equal to the pivot being written from the right end and elements less than the pivot being written from the left end. To do this, each thread needs to know where in shared memory to off load its element. We execute the warp voting function based on a predicate that checks if the element in the register is greater than or equal to the pivot or smaller than the pivot. Threads which have an element greater than or equal to the pivot set the corresponding bit to ‘1’ and to ‘0’ if the element is less than the pivot (Figure 2). If the element in the register is less than the pivot, then the thread needs to find how many of the threads before it have elements less than the pivot and vice-versa. We use a combination of bit shift operations and the _popc(x) function on the integer result of the warp voting operation to accomplish this. The _popc(x) function counts the number of bits set to ‘1’ in the input integer ‘
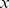
’. For example, if the warp vote integer in binary is 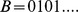, then it is clear that threads 0,2 have elements less than the pivot and threads 1,3 have elements greater than or equal to the pivot. Each thread 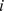 within the warp computes the result 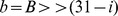. When the _popc(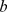) function is applied to the result of this step, it will indicate the position in shared memory at which thread 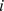 will off-load its element that happens to be less than the pivot (Figure 3). Similarly, 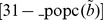 will indicate the position from the right side at which which thread 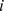 will off load its element that happens to be greater than or equal to the pivot. In the example, thread 3 will bit shift 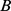 to the right by 29 bits and the result will be 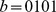. This implies that _popc(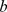) = 2. Therefore thread 3 will off-load its element at the second location from the left in shared memory (Figure 4). Note that the total number of elements in shared memory that are less than the pivot is given by _popc(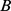). Two global counters, 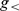 and 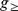, are also maintained to keep track of the total number of elements less than the pivot and total number of elements greater than or equal to the pivot, respectively. These two counters indicate location in the auxiliary array at which the warp writes the incremental results of pivoting from shared memory. Next, the threads write the contents of the shared memory into global auxiliary array with threads whose id is less than _popc(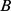) (number of elements that are greater than the pivot) writing from the left side and other threads writing from the right side. This write process requires two coalesced writes, one for elements smaller than the pivot and one for elements greater than or equal to the pivot, into the auxiliary array (Figure 4).

With one warp per query, we must have a minimum of 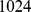 queries to fully utilize GPU resources. If we use one thread block per query, we can reduce the minimum number of queries for full GPU utilization to 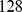. When processing each row of the distance matrix using a thread block, each thread block will have several thread warps (depending on the thread count) that will cooperatively pivot elements. While writing the elements into shared memory, each thread has to determine its relative write position in shared memory with respect to all thread block threads. As a first step, each thread finds its write position with respect to threads in its own warp as described in the previous paragraph. Subsequently, all threads in the thread block are synchronized. An inter warp pre-fix sum step determines the write position of each thread with respect to the entire thread block. The maximum number of thread warps per block is 16 (the max. thread count in a thread block is 512, and the number of threads in a warp is 32). Because of the small number of elements, we use a single thread per thread block to conduct the inter warp prefix sum. This is much more economical than a multi-threaded pre-fix sum.

Once a partition is complete, the output array (auxiliary array) has a left side of length 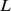 elements, each of which is less than the pivot. The right side is of length 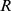 elements each of which is greater than or equal to the pivot. Suppose we need 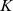 nearest neighbours, and 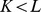, then we need to process only the left hand side. Suppose 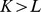, then we keep the left hand side as is, and partition the right hand side to find 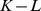 elements. Since the input and auxiliary arrays are swapped at the end of the partition process, in the second case (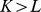), we would have to copy the left hand side from the auxiliary array to the input array. We can avoid copying the data by instead storing a stack of references which indicate the start and end indices and the arrays (auxiliary or input) that the partitions that form the 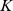 nearest neighbors are to be found. This significantly reduces the memory transactions needed for the operations.

### Distance Calculation

Our implementation supports three distance metrics, namely, Euclidian, Cosine, and Pearson. The Euclidian distance between two vectors 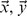 is given by:
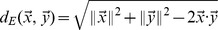



The Cosine distance metric is given by:
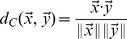



The Pearson distance is given by:
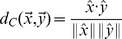



where, 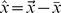 and 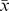 is the mean of the entries in 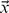. Note that the Pearson distance coefficient is essentially the Cosine distance of the centered data sets.

The process of finding either of these distances requires operations for finding vector means, vector magnitudes, and dot products. For two data sets 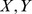, where the columns of 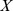 and 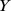 represent the data vectors 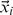 and 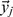 respectively, the 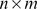 dot products
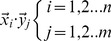
are easily formulated as a matrix product 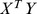. There are very efficient libraries for dense matrix multiplication which can be used for this purpose [35]. The process of finding vector means and vector magnitudes are essentially vector reductions. The thrust library has optimized reduction_by_key which we have used to simultaneously find the vector means and magnitudes of all vectors in 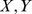.

Finding the Eulcidian and Cosine distances require the same inputs (dot products and norms of 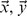). In the first parallel kernel operation, we compute the vector norms of the vectors in 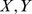 using a combination of a transform iterator and reduction_by_key function. The transform iterator generates the square of individual elements and feeds the result into the reduction_by_key function, which in turn computes the square of the vector norms. The next step is to find the distance. In case the of the Euclidian distance, to find the 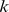 nearest vectors to 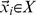 from 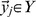 the squared distance formula is given by:
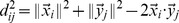



The comparison to find the 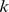 nearest neighbhors of 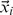 is between the numbers 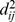. Furthermore, all of the 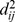 have a common addition factor 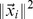. Therefore, it is sufficient to compare the distance metric 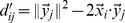. This saves an addition operation. To find the 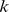 nearest vectors to 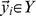 from 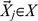, we follow the same process but find the matrix of dot products 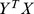 by simply taking a transpose of the matrix 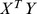 that we computed earlier. The same analysis is valid for Cosine distances as well.

To find the Pearson distance, we need to pre-process the raw data to center it. We use the reduction_by_key to find the vector means. We then center each vector using one thread block per vector. Once the data is centered, the process of finding the distance metric is the same as that of the Cosine distance.

## Results

Our algorithms were written in NVIDIA's CUDA [29] and executed on a Tesla C2050 compute card. We used gcc4.2 with appropriate optimization flags. This card has 448 serial processors and a peak single precision performance of 1.03 TFlops. The RAM memory is 3 GB with a global memory bandwidth of 144 GB/s. We used CUDA 4.2 drivers for our implementation. We performed a comprehensive set of tests against implementations by Garcia et al. [23] and Sismanis et al. [30], both of which provide for *k*-NN. We also benchmarked our algorithm against the nth_element from the standard template library and the GPU-based radix/bucket select implementation of 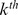-element algorithm by Alabi et al. [33]. The work by Alabi et al., as indicated earlier, is slightly different since they just return the 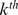 largest or smallest element and not the *k*-NNs. This means that they do not keep track of the *k*-NN elements and therefore perform less work than our algorithm and the implementations by Garcia and Sismanis. For accurate comparison, we downloaded all GPU codes (Sismanis, Garcia, Alabi) and recompiled it using the same compiler and CUDA drivers as the ones we used to compile our code. The test data was a matrix (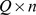, 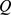 is the number of queries, and 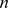 is the size of the corpus data set) of uniformly random floating point numbers with indices. In some tests, indices were left out.

**Figure 5:**
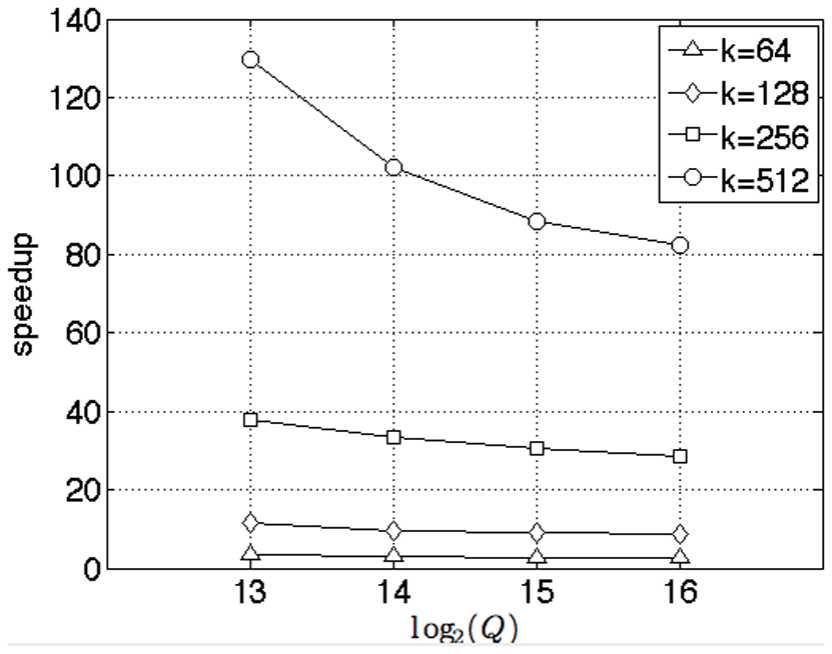
Benchmark against GPU 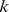 **-NN by Garcia for selection alone with varying size of corpus data (** 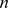) [23]. We set the number of queries 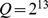 to ensure full GPU utilization. The parameter 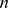 was varied from 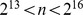. The number of nearest neighbors 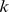 was varied as 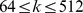. Except for 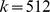, the speedup remains flat with respect to 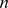. The dramatic gain in performance with 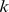 is because the Garcia algorithm shifts its 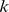-NN stack from shared memory to global memory as 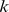 increases. This increases inefficiencies due to uncoalesced memory access. We achieved a speedup 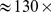 for 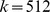 and 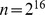.

**Figure 6:**
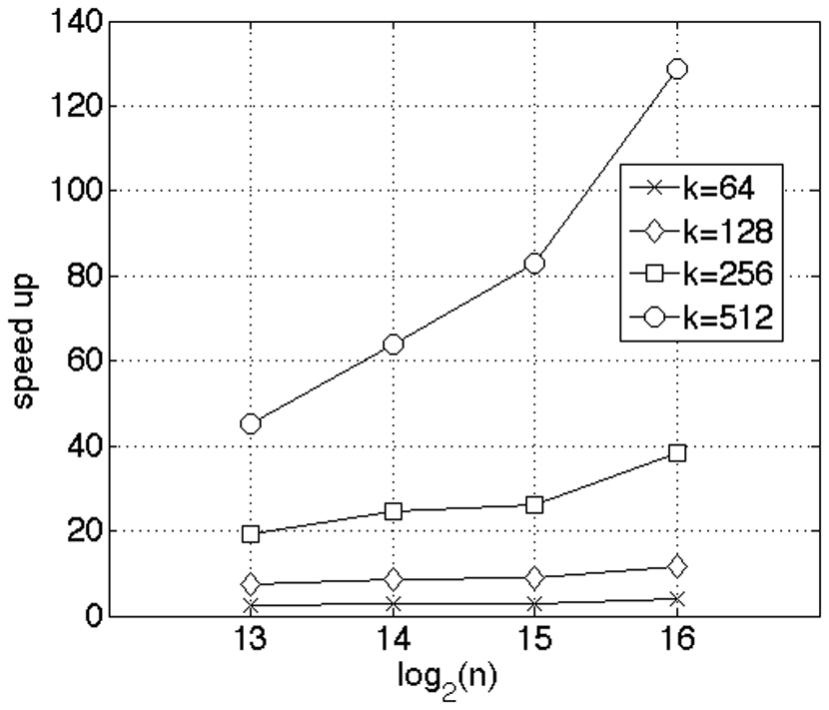
Benchmark of selection alone against GPU 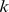 **-NN by Garcia for different query sizes** (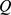). We set 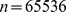. We varied the number of queries as 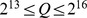. Simultaneously, we varied the number of nearest neighbors as 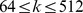. Note that speedup falls as the number of queries 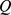 increases. This is because the Garcia algorithm increases GPU utilization as the number of queries is increased. For the largest data sets that fit into GPU memory, corresponding to the level of full GPU utilization by Garcia (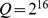) and for the largest number of nearest neighbors (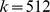), our algorithm is 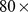 faster.

For large data sets, the code developed by Garcia et al. [23], batches execution of multiple queries. For large 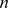, the size of the batches (i.e., the number of queries) can cause underutilization of GPU resources. We chose parameters in our data sets 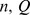 so that the algorithm by Garcia operated at its full potential. Figure 5 shows the results of the benchmarks where we kept 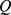 constant and varied the parameters 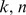. We set the number of simultaneous queries 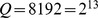. We varied the number of closest neighbors as 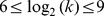. Also, we varied the size of the corpus data as 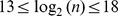. We only benchmarked the search part of the algorithm, since the method for distance calculation is virtually identical between the two methods. Figure 6 shows the performance advantage of our algorithm. There is dramatic gain in speedup when 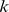 grows. As 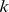 grows, Garcia's algorithm shifts its 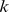-NN stack from shared memory to global memory and is saddled with all the associated pitfalls, such as un-coalesced memory access. In Figure 6, we illustrate the effect of changing the number of queries. In this example, we kept the size of the corpus at 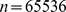 and changed the number of queries as 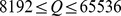. Simultaneously, we changed the number of selections as 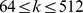. For small queries, Garcia's method underutilizes the GPU. As we increase the number of queries, the GPU utilization increases, and therefore, we see a decrease in speedup from the peak. For the largest sizes of 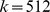 and 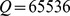 that fit in our GPU memory, our method is 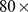 faster.

**Figure 7:**
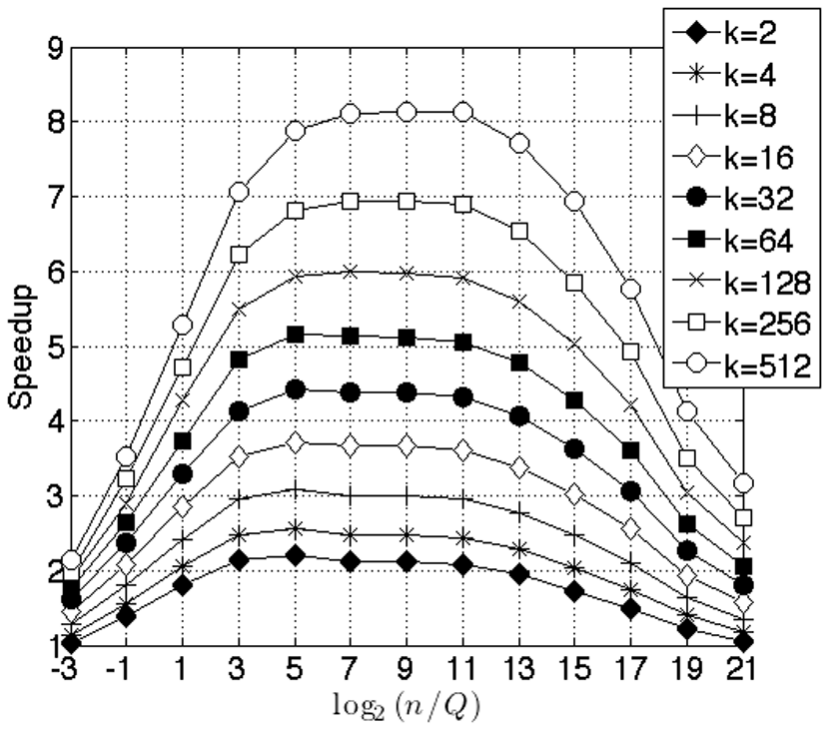
Benchmark against work by Sismanis et al. [30]. These tests were performed for elements that contained both values and indices. In this benchmark we varied 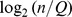 as 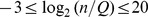. We kept the product 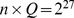. We simultaneously varied 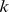 as 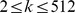. Performance peaks at 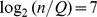 stays flat untill 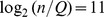 and starts falling at 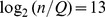. The decrease in performance corresponds to the number of queries 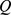 falling below 128 where our method underutilizes GPU resources.

For the benchmarks against work by Sismanis (Figure 7), we compared the selection method alone. We kept the product 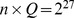 constant, where 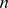 is the number of data corpus elements (columns in the distance matrix) and 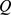 is the number of query elements (rows in the distance matrix). We plotted our speedup against the ratio 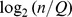. We ran a thousand trials for each evaluation and averaged the results. As seen in Figure 7, the performance gains for various 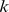 rapidly rise with 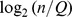, reach a saturation level, and then fall for large values of 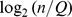. The degradation of performance occurs at 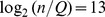. This point corresponds to 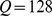, i.e., where the number of thread blocks falls below 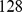. At this stage, our algorithm is not able to fully utilize GPU resources. We show in a subsequent test that our algorithm saturates the GPU only when the thread block count exceeds 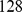. The performance advantage grows with 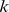. We were able to benchmark only up to 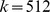 as the Sismaniss implementation cannot handle 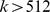. With increased GPU RAM, we can handle larger distance matrices and therefore, have more queries 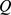 for large values of 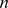. For example, if we had a GPU with 18 GB of available RAM instead of the current 2 GB (usable), we would be easily able to handle distance matrices of sizes 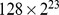. This would push the point of performance degradation to a point given by 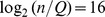. Consequently, we expect our algorithm to perform even better on fused GPU-CPU architectures that have unified memory access to main CPU RAM. Such processors will become mainstream in the next 2–3 years.

**Figure 8:**
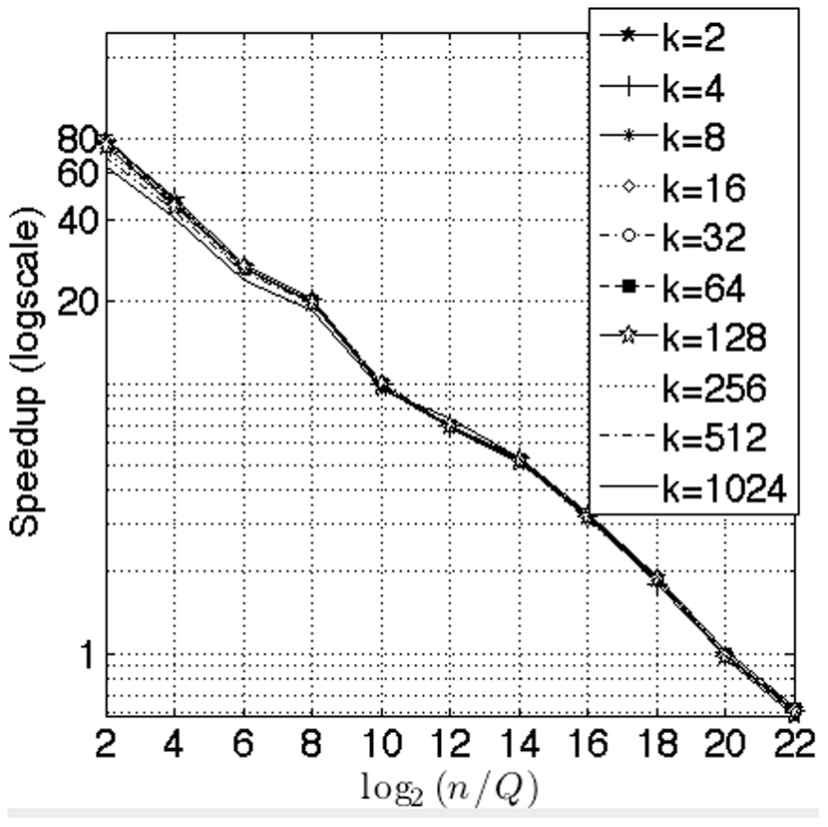
Benchmark against work by Alabi et al. [33]. Here the benchmark is comparing the speedup of selecting 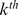 element vs selection of 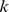-NN using our algorithm. These tests were performed for elements with values alone. We kept the product 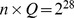 and varied 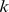 as 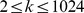. Simultaneously, we varied 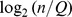 as 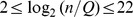. There is a dramatic fall in performance gain because the method by Alabi increases GPU utilization as 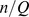 increases. It reaches saturation at 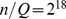 [33]. Our algorithm starts underutilizing GPU resources at 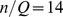. Even when Alabi et al. is saturated and our algorithm is significantly underutilizing GPU resources, we are 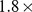 faster. Once again, with a larger GPU RAM, our algorithm would perform significantly better.

Our third set of benchmarks were against the radix/bucket select-based 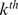 statistic algorithm implemented by [33]. This implementation takes one query at a time. Therefore, our comparison tests involved initially loading the entire distance matrix into GPU RAM and then invoking the radix select algorithm one query at a time. This approach adds kernel launch overhead to the time for calculation. However, the typical kernel launch overhead for the GPU in our tests is 5.7 micro seconds which is nearly two orders of magnitude smaller than the time required for task execution. Figure 8 shows the results of our benchmarks. For this battery of tests, we kept the product 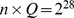 constant. Once again, we ran a thousand trials for each evaluation and averaged the results. For low values of 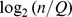, our approach is significantly better. This is because in this regime, the implementation by [33] underutilizes GPU resources. As the ratio 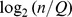 increases, our performance advantage decreases. At 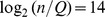, our algorithm starts underutilizing GPU resources because the number of thread blocks in operation falls below 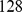. The method of [33] achieves GPU saturation only at 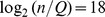 (i.e., 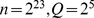). Even at this point, our algorithm is slightly faster (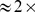), even though it is underutilizing GPU resources and is performing a significantly larger amount of work keeping track of 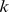-NNs instead of finding just the 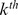 statistic.

**Figure 9:**
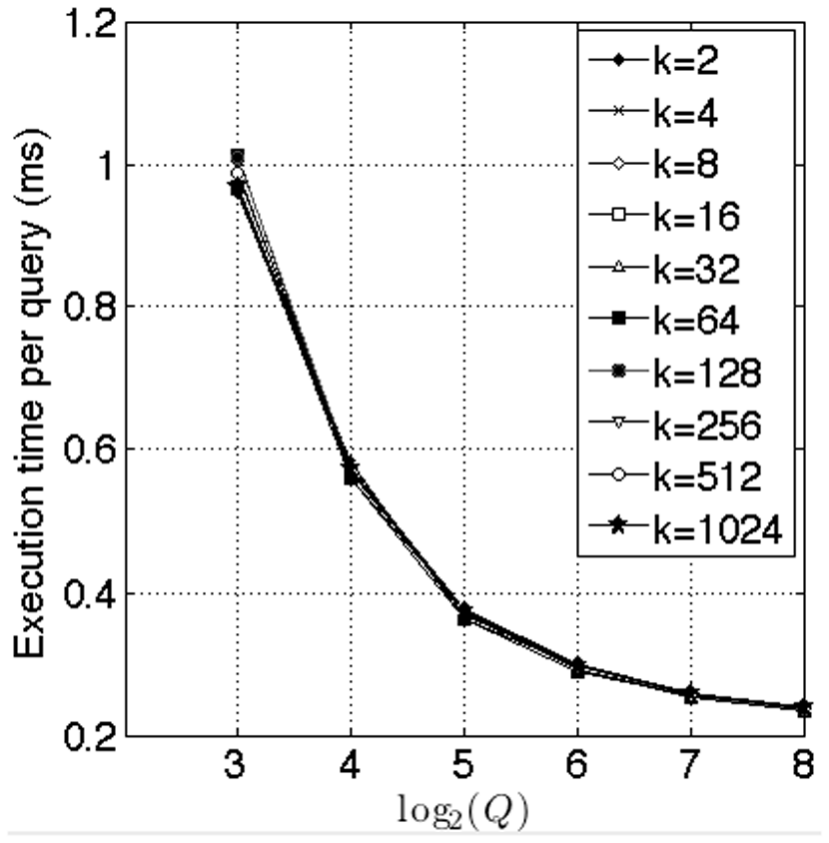
Effect of varying the number of queries 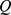. In this benchmark, we kept 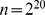 and varied the number of queries as 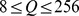. As can be seen from the graph for various values of 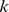, the time per query decreases with the increase in 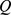 and roughly flattens out at 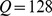.

Figure 9 illustrates our tests to investigate GPU saturation. In these tests, we kept 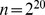 constant and varied 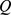, the number of queries from 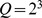 to 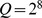. For various values of 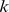, it can be seen that the time per query drops with the 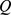 and flattens out at 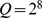. Since we are utilizing one thread block per query, we should have at least 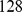 queries to fully utilize GPU resources.

**Figure 10:**
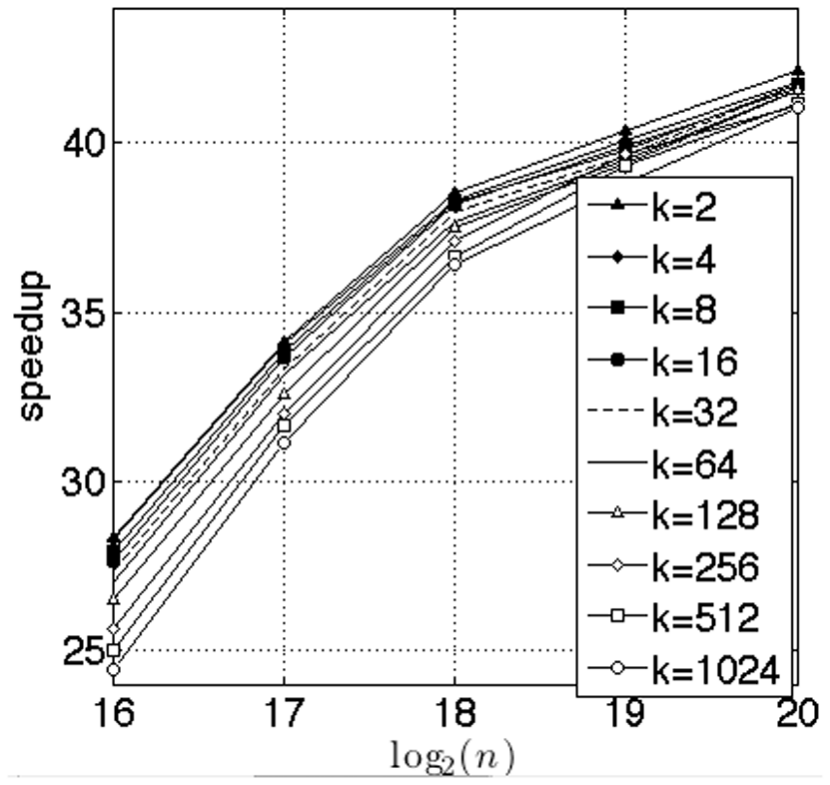
Benchmark against 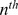 **element.** This benchmark is against a single core of the CPU. As can be seen, the performance degrades slightly with larger values of 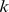. The number of queries were set to 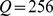. We ran up to 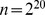 and achieved around 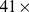 speedup. As can be seen from the figure, the speedup graph has not been saturated for the largest values of 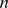 that can be accommodated on our GPU.

We benchmarked our selection algorithm against the Standard Template Library nth_select. Just as in [33], the nth_select algorithm works on a single query. We compared our implementation with a single threaded nth_select working one query at time. The data was loaded into memory ahead of time and queries were processed in a for loop. We used a Intel Core i5-3750K running at 3.8 GHz. This CPU has 4 cores with 32 KB L1 cache, 256 KB L2 cache, and 6 MB L3 cache shared between the 4 cores. Figure 10 shows the results of the benchmark. In this test, we varied the parameter 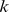 as 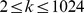 and the parameter 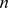, as 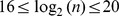. The speedup increases with 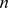 and, as can be seen in the figure does not reach saturation even at 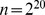. There is a slight degradation with the increase in 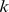, which is obvious since the larger 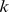 entails keeping track of a larger list of elements. In our tests, we achieved around 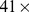 speedup for the largest 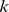 with the largest data sets that could be accommodated in our GPU RAM.

Finally, in Table 2, we show variation in time for different data sets. Time for execution is dependent on the data set. Therefore, we ran about 1000 trials for each setting and measured the mean and standard deviation. Table 2 lists the results. It appears that the standard deviation in timing decreases as 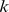 increases. Overall the standard is less than 0.5%.

**Table 2:** Execution time variation.

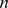	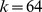		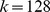		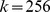		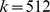	
	mean	%std	mean	%std	mean	%std	mean	%std
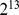	138.03	0.37	144.4	0.27	151.36	0.24	159.35	0.31
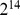	163.55	0.33	170.04	0.311	177.27	0.25	185.91	0.27
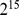	208.83	0.29	215.3	0.37	222.97	0.28	232.10	0.245
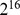	288.44	0.29	294.85	0.28	302.34	0.27	311.8	0.26

In this study, we kept the number of queries 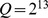 and varied 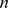 and 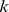 to study the mean and standard deviation of execution time over 1000 trials for each data set (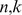).

## Discussion

We have successfully developed and implemented a multi-query 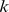-NN algorithm on a GPU. Our algorithm is an implementation of the Quick Select method and uses the architectural advantages of modern GPUs. For corpus data that fit in GPU memory, based on our benchmarks, our algorithm outperforms all current state-of-the art methods. There are, however, many limitations to our method. To achieve full GPU saturation, we cannot have the number of queries 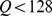, i.e., we need to have at least 128 thread blocks running simultaneously. Given the roughly 2 GB available memory, this restricts the number of elements in the corpus data to 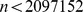. With the newer fused GPU-CPU processors, with unified RAM (up to 64 GB), we will be able to handle much larger data sets. We are also looking at batch execution with data partitioning for large corpus data that do not fit into GPU memory. We may be able to overlap computation with data transfer to and from the CPU RAM to reduce overall execution time. Batch execution will obviously require merging of results between executions and additional memory to store intermediate results. We believe there will be significant opportunities for optimization based on the number of queries, corpus data size, and the dimension of vectors in the corpus data. This research will be explored in subsequent publications.
